# A focus on capacitive cation sensing under flow: play it again SAM

**DOI:** 10.1039/d4sc90214a

**Published:** 2024-10-30

**Authors:** Debapriya Gupta, Amar H. Flood

**Affiliations:** a Department of Chemistry, Indiana University 800 East Kirkwood Avenue Bloomington IN 47405 USA aflood@iu.edu

## Abstract

We highlight an article by Patrick *et al.* (S. C. Patrick, R. Hein, P. D. Beer and J. J. Davis, *Chem. Sci.*, 2024, https://doi.org/10.1039/D4SC05271D) that integrates capacitance spectroscopy with a continuous flow assay to achieve reagentless electrochemical sensing. Injection of analytes leads to ion recruitment by the self-assembled monolayer (SAM) of benzo-15-crown-5 receptors generating spikes in the sensorgram. Injecting the supporting electrolyte regenerates the vacant receptor, returning the value to baseline. Binding and debinding kinetic signatures also emerge. The continuous and sensitive reporting of interfacial host–guest binding in real-time follows the precision of a well-composed melody that can be played again and again.

## Abstract 2

We highlight an article by Patrick *et al.* (*Chem. Sci.*, 2024, https://doi.org/10.1039/D4SC05271D) that integrates capacitance spectroscopy with a continuous flow assay to achieve reagentless electrochemical sensing.

## Graphical abstract

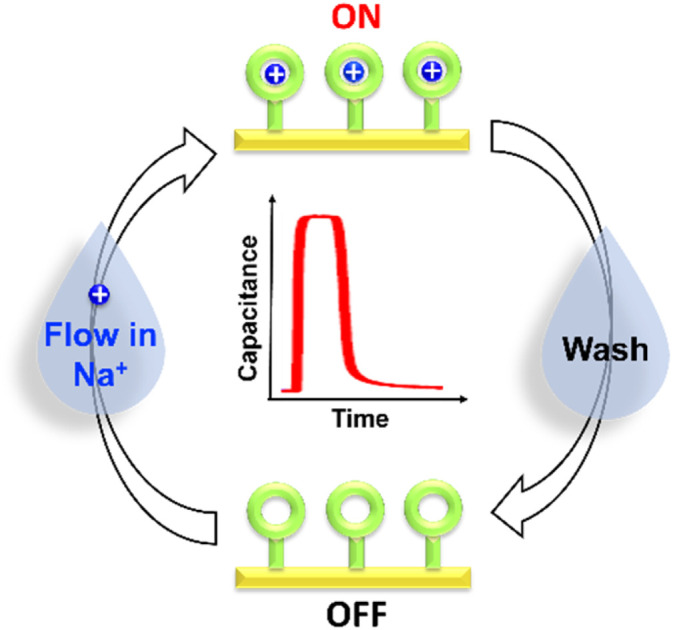

Surface-mounted supramolecular receptors offer excellent opportunities for studying host–guest chemistry in interfacial environments,^1^ mimicking biological molecular recognition events. Such receptors have the potential to be used in sensing since they can exhibit an amplified response to analytes compared to receptors in solution.^2^ However, constant monitoring is necessary for real-world sensing applications. Techniques for studying the binding interactions between surface-bound receptors and analytes are still relatively scarce,^3^ and mostly applied to the study of biomolecules.^4^ Electrochemical impedance spectroscopy (EIS) is one of the methods by which interfacial recognition events of surface-immobilized receptors can be studied.^3^ Traditional faradaic impedimetric ion sensing can detect ion recruitment by self-assembled monolayers (SAMs) of supramolecular receptors.^3^ However, the need for a redox-probe imposes multiple restrictions on the measurement process, including solubility of the probe in organic media and the electroactivity of analyte anions, and suffers from baseline instability. Capacitance spectroscopy has been used to detect ion binding by surface-immobilized supramolecular receptors based on the variation in monolayer capacitance after the addition of charged guests.^5–7^ This non-faradaic approach is preferable for applications due to its clear synthetic and experimental benefits, such as not requiring redox probes, and maintaining a stable baseline.

In recent work, Patrick *et al.* demonstrate the use of capacitance spectroscopy for continuous and real-time flow detection of cations (https://doi.org/10.1039/D4SC05271D).^8^ A modified benzo-15-crown-5 was used as the supramolecular receptor, and the SAMs of the receptor were formed on gold electrodes (Fig. 1a). The capacitance of the film was measured before and after exposure to alkali metal cations in a static solution. A distinct increase in the capacitance value was observed after the exposure, attributed to the higher dielectric constant of the thin film after ion recruitment. Additionally, hydration-induced expansion of the electrode's surface area contributed to the increased capacitance values.

**Fig. 1:**
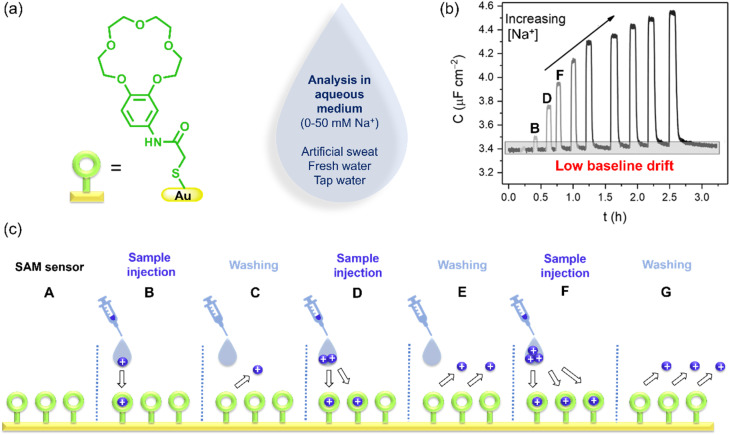
(a) The receptor is a modified benzo-15-crown-5 that is immobilized on a gold electrode surface of a sensor for analysis of sodium (Na^+^) and other cations. (b) Non-faradaic capacitance response, *C*, of the surface-mounted receptor toward increasing Na^+^ concentrations. (c) Cartoon showing the ion binding and debinding process by the surface-immobilized receptor under continuous flow (reproduced from ref. 8 with permission from the Royal Society of Chemistry).

This system was integrated with a 3D-printed flow cell to afford continuous real-time sensory readouts for monitoring cation levels (Fig. 1b). Introduction of the sample aliquot (Na^+^) leads to ion recruitment by the benzo-15-crown-5 receptors, generating a spike in the capacitance value in the sensorgram (see Fig. 1b and c, spike B). In the next step (C), supporting electrolyte is injected to regenerate the vacant receptor thereby returning the film capacitance to baseline values. Long-term sensing experiments, with measurements conducted for fifteen additions and over the course of 54 hours, demonstrated minimal difference in capacitance readouts and stable baseline values. One clear advantage of the flow system is the opportunity to repeat binding events (Fig. 1b and c) over an extended period of time.

The crown-based sensor is most sensitive to Na^+^ with the trend in selectivity following the size complementarity of the receptor under both static and flow conditions. The binding constant values for Li^+^, Na^+^ and K^+^, determined using a Langmuir model, mirror the binding pattern of the receptor in solution.^9^

The sensitivity of the sensor towards Na^+^ exceeds that of the commercial ion selective electrodes (ISEs) used for in-the-field Na^+^ testing.^10,11^ The method also shows low drift, which is known to be a problem for commercial ISEs. Another advantage of the flow system is the quick regeneration of vacant receptors after ion binding. This behavior removes the necessity for re-calibration, which is typically required to restore the baseline after each measurement with ISEs. The accuracy of the baseline recovery after washing is impressive and is reproduced with a range of different cations and measurement times ranging from 5–54 hours.

The team showcased the method for sensing Na^+^ in real-world samples. Multiple samples of the simulated sweat matrix containing physiologically relevant concentrations of Na^+^ (typically between 10–100 mM) were tested using this method. The results showed excellent agreement between absolute and measured Na^+^ values in all cases, including samples of fresh water and tap water tested using this method.

The study highlights specific points regarding the ion recruitment process of supramolecular receptors at the interface. The binding constants determined for the larger cations Rb^+^ and Cs^+^ were higher than expected. This difference pointed the authors to the possibility of 2 : 1 host : guest sandwich complexes.

The capacitance responses recorded during continuous measurements also displayed kinetic signatures for the association and dissociation processes. Upon analyte injection, the increase in capacitance reflects the kinetics of binding, followed by a plateau and a return to baseline values in the washing cycle indicating dissociation of the ion from the receptor. Patrick *et al.* correlated the temporal fingerprints with ionic size. Smaller cations (Li^+^ and Na^+^) exhibit rapid and reversible binding to the receptor. However, for larger cations, a slow dissociation process was observed. The rates of association and dissociation of the cations (Li^+^, Na^+^, K^+^, Rb^+^, Cs^+^ and NH_4_^+^) were modeled and the calculated rate constants of association were broadly in agreement with the binding affinity trends. The rates of dissociation were significantly slower for larger cations, which again points to the possibility of 2 : 1 sandwich complex formation. Kinetic analyses of small molecule host–guest recognition at interfaces are rare and Patrick *et al.* have undertaken one of the pioneering efforts in this direction.

In summary, the article by Patrick *et al.* introduces a detection method to constantly monitor ion concentrations by binding to receptor-modified interfaces in real time. The method is reagentless and eliminates persistent problems faced by the incumbent ISEs (recalibration and drift). The method can also be extended to monitor other receptor–ion combinations. This approach is crucial for supramolecular chemists seeking to understand the ion-recruitment process and the selectivity of surface-immobilized receptors. Supramolecular receptors used in sensing devices are often incorporated inside membrane sensors^12^ or in the conductive channels of polymer-based transistor sensors.^13^ Despite their utility in sensing, these devices offer little opportunity to study the host–guest recognition event on a molecular level. This is where the method by Patrick *et al.* proves to be extremely valuable. The possibility of determining the thermodynamics and kinetics of interfacial ion association or dissociation is exciting and is expected to provide new insights into the world of interfacial molecular recognition and self-assembly.

## Author contributions

D. G. and A. H. F. co-wrote the manuscript.

## Conflicts of interest

There are no conflicts of interest to declare.
